# Fibrinolysis protease receptors promote activation of astrocytes to express pro-inflammatory cytokines

**DOI:** 10.1186/s12974-019-1657-3

**Published:** 2019-12-06

**Authors:** Paola Pontecorvi, Michael A. Banki, Carlotta Zampieri, Cristina Zalfa, Pardis Azmoon, Maria Z. Kounnas, Cinzia Marchese, Steven L. Gonias, Elisabetta Mantuano

**Affiliations:** 1 The Department of Pathology, University of California San Diego, 9500 Gilman Drive, La Jolla, CA 92093-0612 USA; 2 The Department of Experimental Medicine, Sapienza University of Rome, 00161 Rome, Italy; 3 The Department of Chemical Sciences and Technologies, Tor Vergata University of Rome, 00133 Rome, Italy

**Keywords:** Astrocyte, Microglia, Inflammation, Plasminogen, Tissue-type plasminogen activator, Urokinase-type plasminogen activator, uPAR, α-Enolase, Protease-activated receptor

## Abstract

**Background:**

Astrocytes contribute to the crosstalk that generates chronic neuro-inflammation in neurological diseases; however, compared with microglia, astrocytes respond to a more limited continuum of innate immune system stimulants. Recent studies suggest that the fibrinolysis system may regulate inflammation. The goal of this study was to test whether fibrinolysis system components activate astrocytes and if so, elucidate the responsible biochemical pathway.

**Methods:**

Primary cultures of astrocytes and microglia were prepared from neonatal mouse brains. The ability of purified fibrinolysis system proteins to elicit a pro-inflammatory response was determined by measuring expression of the mRNAs encoding tumor necrosis factor-α (TNF-α), interleukin-1β (IL-1β), and chemokine (C-C motif) ligand 2 (CCL2). IκBα phosphorylation also was measured. Plasminogen activation in association with cells was detected by chromogenic substrate hydrolysis. The activity of specific receptors was tested using neutralizing antibodies and reagents.

**Results:**

Astrocytes expressed pro-inflammatory cytokines when treated with plasminogen but not when treated with agonists for Toll-like Receptor-4 (TLR4), TLR2, or TLR9. Microglia also expressed pro-inflammatory cytokines in response to plasminogen; however, in these cells, the response was observed only when tissue-type plasminogen activator (tPA) was added to activate plasminogen. In astrocytes, endogenously produced urokinase-type plasminogen activator (uPA) converted plasminogen into plasmin in the absence of tPA. Plasminogen activation was dependent on the plasminogen receptor, α-enolase, and the uPA receptor, uPAR. Although uPAR is capable of directly activating cell-signaling, the receptor responsible for cytokine expression and IκBα phosphorylation response to plasmin was Protease-activated Receptor-1 (PAR-1). The pathway, by which plasminogen induced astrocyte activation, was blocked by inhibiting any one of the three receptors implicated in this pathway with reagents such as εACA, α-enolase-specific antibody, uPAR-specific antibody, the uPA amino terminal fragment, or a pharmacologic PAR-1 inhibitor.

**Conclusions:**

Plasminogen may activate astrocytes for pro-inflammatory cytokine expression through the concerted action of at least three distinct fibrinolysis protease receptors. The pathway is dependent on uPA to activate plasminogen, which is expressed endogenously by astrocytes in culture but also may be provided by other cells in the astrocytic cell microenvironment in the CNS.

## Background

In the CNS, microglia and astrocytes regulate innate immunity and neuro-inflammation [1, 2]. By secreting cytokines and other extracellular mediators, these cells establish crosstalk with one another and with local neurons and also recruit inflammatory cells from the blood. Microglia express a large continuum of Pattern Recognition Receptors (PRRs), including diverse members of the Toll-like Receptor (TLR) family [3, 4]. By contrast, astrocytes express a more limited repertoire of PRRs, including only low levels of specific TLRs, such as TLR3 [5–7]. Thus, understanding the signals that lead to astrocyte activation and contribute to neuro-inflammation remains an important problem.

Proteins that function in hemostasis have been implicated in innate immunity and in the regulation of inflammation, including Tissue Factor, Fibrinogen, and Protein C [8–11]. The fibrinolysis system is the arm of hemostasis that systematically lyses blood clots and maintains patency in uninjured blood vessels [12]. The principal activators of fibrinolysis are tissue-type plasminogen activator (tPA) and urokinase-type plasminogen activator (uPA), both of which convert plasminogen (Plg) into plasmin (PM), which degrades fibrin [12, 13]. In recent years, cellular receptors for fibrinolysis proteases and their zymogens have been identified. A family of structurally diverse but functionally similar receptors for Plg have been characterized [14]. These receptors, which in many cases also bind tPA, amplify the rate of Plg activation, localize PM activity to the outer cell membrane, and participate in pathways that result in cell-signaling [14]. The urokinase receptor (uPAR) is a glycosylphosphatidylinositol (GPI)-anchored receptor that increases the catalytic efficiency of Plg by uPA and also associates with transmembrane proteins to form a receptor complex that signals in response to vitronectin and uPA [15]. tPA interacts with a distinct receptor complex that includes the N-methyl-D-aspartate (NMDA) receptor and Low-Density Lipoprotein Receptor-related Protein-1 (LRP1) to trigger cell-signaling and regulate innate immunity [16, 17]. Finally, it has been shown in studies mainly with monocytes and macrophages that PM directly cleaves GPCRs in the Protease-activated Receptor (PAR family) such as PAR-1 and PAR-2 to regulate innate immunity [18–21]. Cleavage of PAR-1 by PM also has been shown in astrocytes [22]. In these cells, PAR-1 cleavage activates phosphoinositide-3 kinase (PI3K) and induces expression of transforming growth factor-β3 [22]. The function of fibrinolysis receptors in astrocytic activation and their possible effects on neuro-inflammation was the topic of the current study.

We compared microglia and astrocytes isolated from mouse pups and showed that both cell types demonstrate increased expression of pro-inflammatory cytokines, including TNFα, interleukin-1β (IL-1β), and CCL2 when treated with Plg. Plg was active in microglial cultures only when added together with plasminogen activator to generate PM. By contrast, neonatal mouse astrocytes (N-astrocytes), which were not responsive to activators of TLR2, TLR4, or TLR9, expressed increased levels of cytokines in response to Plg alone. The responsible pathway required uPA, which was expressed endogenously by N-astrocytes, together with a system of fibrinolysis receptors that included the Plg receptor, α-enolase, uPAR, and PAR-1. Despite the well-documented role of uPAR in cell-signaling [15], including in astrocytes [23], uPAR apparently functioned only to facilitate Plg activation in the pathway that resulted in pro-inflammatory cytokine expression. The amino terminal fragment of uPA (ATF), which replicates most of the cell-signaling activity of uPA [24–26], functioned as an antagonist of this pathway. Overall, our results define a multicomponent, linear pathway, involving three distinct fibrinolysis receptors, that couples Plg to astrocytic activation. Although in the adult brain, astrocytes may express decreased levels of uPA, compared with cultured N-astrocytes, astrocytes also may become loaded with uPA that is transferred from neurons [23]. These results suggest novel opportunities for pharmacological modulation of neuro-inflammation.

## Materials and methods

### Proteins and reagents

Glu-Plasminogen was purified from human plasma as previously described [27]. Human tPA, which is produced in CHO cells and 95% in the two-chain form, was from Molecular Innovations. Lipopolysaccharide (LPS) serotype 055:B5 from *E*. *coli* was from Sigma-Aldrich. The TLR2 ligand, lipoteichoic acid (LTA) from *S*. *aureus* and the TLR9 ligand, ODN 1826, were from InvivoGen. Amiloride (AMD) was from Sigma-Aldrich. Mouse uPAR-specific antibody (cat. AF534) and control IgG (cat. AB105C) were from R&D Systems. α-Enolase-specific polyclonal antibody was from Invitrogen (cat. 3810T). Rabbit polyclonal antibody that targets the C-terminus of actin was from Sigma-Aldrich (cat. A2066). ε-Aminocaproic acid (εACA) was from MP Biomedicals. Aprotinin was from PanReac AppliChem. SCH 79797 was from Cayman Chemicals. The plasmin-specific chromogenic substrate, H-D-Val-Leu-Lys-p-nitroanilide (S-2251) and mouse uPA ATF were from Molecular Innovations.

### Cell culture

Microglia and N-astrocytes were isolated from C57BL/6J mouse pups [28]. In brief, brains were harvested from postnatal day 1–6 mice. The cortices were dissected from the forebrain, and the surrounding meninges were removed. Intact cortices were mechanically and enzymatically dissociated using the Neural Tissue Dissociation Kit P (Miltenyi Biotec). Mixed glial cultures were established in Dulbecco's modified Eagle's medium/F-12 (DMEM/F-12) supplemented with GlutaMAX (Gibco), 10% fetal bovine serum (FBS, Gibco), and 100 units/ml Antibiotic-Antimycotic (Gibco). After culturing for 10–14 days, microglia was harvested by shaking at 200 rpm for 30 min at 37 °C. The floating cells were collected by centrifugation and re-plated at 3 × 10^5^ cells/well. Oligodendrocytes were removed by an additional 6 h of shaking. Then, N-astrocytes were collected by trypsinization and subsequent centrifugation and re-plated at 3.5 × 10^5^ cells/well on Poly-D-Lysine hydrobromide-coated wells in DMEM-High Glucose supplemented with 10% FBS and 100 units/ml Antibiotic-Antimycotic. Experiments were performed within 24 h of completing the isolation procedure for microglia and within 48 h of completing the isolation procedure for N-astrocytes.

Bone marrow cells were isolated from the femurs of 16-week-old wild-type C57BL/6J male mice, as previously described [29]. Cells were plated in non-tissue culture-treated dishes and cultured in DMEM/F-12 medium containing 10% FBS and 20% L929 cell-conditioned medium for 8 days. Non-adherent cells were eliminated on day 10. Adherent cells included > 95% bone marrow-derived macrophages (BMDMs) as determined by F4/80 and CD11b immunoreactivity. All reagents used in this study were tested for their effects on viability of cells and had no effect as determined by MTT assay (Invitrogen).

### RT-qPCR

In cytokine expression experiments, microglia and N-astrocytes were cultured in serum-free medium (SFM) for 30 min and then treated simultaneously for 6 h with various proteins and reagents, including tPA (12 nM), Plg (0.2 μM), LPS (0.1 μg/mL), LTA (1.0 μg/mL), ODN 1826 (1.0 μg/mL), aprotinin (33 units/mL), SCH 79797 (2 μM), amiloride (100 μM), uPAR-specific antibody (1 μg/mL), α-enolase-specific antibody (10 μg/mL), εACA (10 mM), or the uPA ATF (concentration as indicated). BMDMs were serum-starved for 30 min and then treated for 3 h with tPA (12 nM) plus Plg (200 nM), LPS (0.1 μg/mL), LTA (1.0 μg/mL), ODN 1826 (1.0 μM), or vehicle (20 mM sodium phosphate, 150 mM NaCl, pH 7.4). Total RNA was isolated using the NucleoSpin RNA kit (Macherey-Nagel) in the presence of DNAse and reverse-transcribed into cDNA using the iScript cDNA Synthesis Kit (Bio-Rad). RT-qPCR was performed with TaqMan gene expression products and an AB Step One Plus Real-time PCR System (Applied Biosystems). No-reverse transcriptase controls were performed routinely. The primer-probe sets were GAPDH (Mm99999915_g1), RPL13A (Mm01612986_gH), SDHA (Mm01352366_m1), TNFα (Mm00443258_m1), IL-1β (Mm00434228_m1), and CCL2 (Mm00441242_m1). The relative change in gene expression was calculated using the 2^−ΔΔCt^ method and GAPDH mRNA as a normalizer.

### Immunoblot analysis

Microglia and astrocytes were extracted in RIPA buffer (PBS with 1% Triton X-100, 0.5% sodium deoxycholate, 0.1% SDS) supplemented with Halt Protease and Phosphatase Inhibitor Cocktails (Thermo Scientific). The protein concentration in cell extracts was determined by DC Protein Assay (Bio-Rad). An equivalent amount of cellular protein was subjected to SDS-PAGE and electro-transferred to polyvinylidene fluoride membranes. The membranes were blocked with 5% nonfat dry milk in 20 mM Tris-HCl, 150 mM sodium phosphate, pH 7.4 with 0.1% Tween 20 (TBS-T buffer) and incubated with primary antibodies from Cell Signaling Technology, including antibodies that target phospho-IκBα (Ser32) (1:1000, cat. 2859), IκBα (1:1000, cat. 9242), and β-actin (1:5000, cat. 3700), followed by horseradish peroxidase conjugated secondary antibodies (Jackson ImmunoResearch). Immunoblots were developed using ProSignal Pico and Femto ECL Reagent substrate (Prometheus) and imaged using the Azure Biosystems c300 imaging system.

### PM activity assay

N-astrocytes and microglia were seeded in 48-well plates (1 × 10^5^ cells/well). Other wells were coated with FBS in the absence of cells. After 48 h, cells were washed 3 times and the medium replaced with HBSS with Mg^+^ and Ca^2++^ (Gibco), 10 mM HEPES and 1 mg/mL bovine serum albumin (BSA), pH 7.4 (H-buffer). Plg, tPA, and/or amiloride were added as indicated so the final volume was 180 μL. The concentrations for tPA and amiloride were 12 nM and 100 μM, respectively. The concentration of Plg was 0.2 μM. S-2251 (0.2 mM) was added 6 h later. S-2251 hydrolysis was detected by monitoring the absorbance at 405 nm as a function of time using a SpectraMax M2 microplate reader.

### Plg binding to N-astrocytes

Human Glu-Plg was labeled with Alexa Fluor 594 (Invitrogen). Labeling reactions were carried out according to the manufacturer’s instructions. The total reaction mixture was transferred to a BioGelP-30 fine size exclusion purification resin. PBS was used as an elution buffer to separate Plg-Alexa Fluor 594 conjugate (Plg-594) from free dye. Purified Plg-594 was quantified by measuring the absorbance at 280 nm and 590 nm following manufacturer’s instructions. N-astrocytes were plated at 1.3 × 10^4^ cells/well in 96-well plates in DMEM-high glucose supplemented with 10% FBS and 100 units/ml Antibiotic-Antimycotic. The cells were then transferred to H-buffer. Plg-594 was incubated with the cells at 4 °C with gentle agitation for 4 h. Binding studies were performed at 4° to minimize Plg activation, which may be associated with the generation of new Plg binding sites [14]. εACA was included in some wells to distinguish specific and non-specific binding [30]. The cells were then washed 3 times with H-buffer. Cell-associated Plg-594 was recovered in PBS plus εACA and quantified by fluorescent emission at 617 nm using an excitation wavelength of 590 nm and a cut-off filter of 610 nm in a SpectraMax M2 microplate reader.

### Statistics

Statistical analysis was performed using GraphPad Prism 5.0 (GraphPad Software Inc.). All results are expressed as the mean ± SEM. RT-qPCR data were analyzed by one-way ANOVA followed by Tukey’s multiple comparison test (*p < 0.05, **p < 0.01, ***p < 0.001).

## Results

### N-astrocytes express pro-inflammatory cytokines in response to Plg but not in response to multiple TLR ligands

**Fig. 1:**
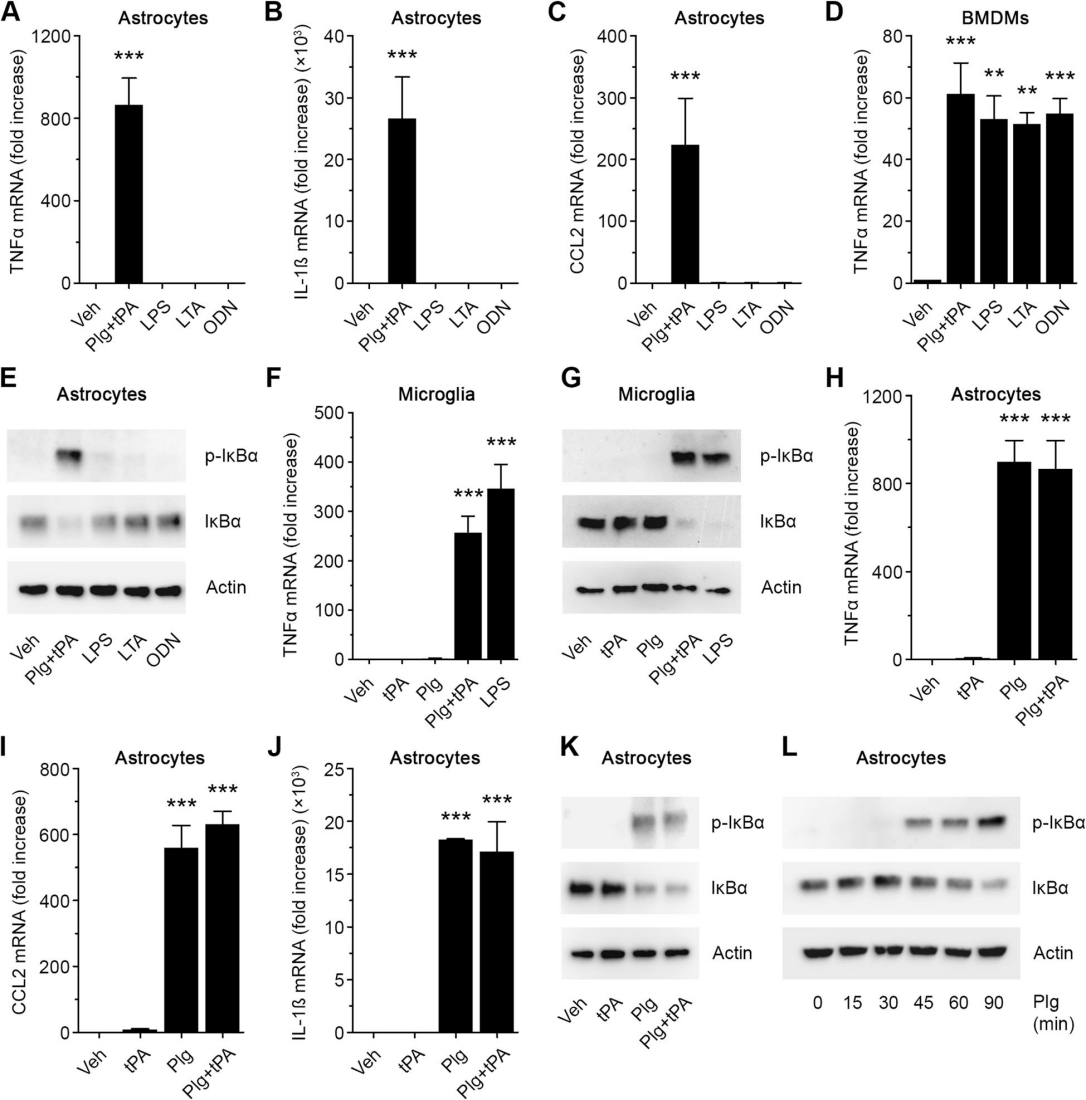
Plg induces expression of cytokines independently of tPA in N-astrocytes **a**–**c** N-astrocytes were serum-starved for 30 min and then treated for 6 h with plasminogen (Plg, 0.2 μM) plus tPA (12 nM), lipopolysaccharide (LPS, 0.1 μg/mL), lipoteichoic acid (LTA, 1.0 μg/mL) or oligodeoxynucleotides (ODN, 1 μM). Expression of the mRNAs encoding TNFα, IL-1β and CCL2 was determined (mean ± SEM; *n* = 3; ***p < 0.001; one-way ANOVA with Tukey’s post hoc test. Statistical analysis is relative to the vehicle control). **d** BMDMs were treated for 3 h with Plg plus tPA, LPS, LTA or ODN. RT-qPCR was performed to compare mRNA levels for TNFα (mean ± SEM; *n* = 3; ***p < 0.001, **p < 0.01; one-way ANOVA with Tukey’s post hoc test). The presented results show the “fold increase” in mRNA expression compared with cultures treated with vehicle. **e** Serum-starved N-astrocytes were treated for 1 h with Plg plus tPA, LPS, LTA (1.0 μg/mL), (1 μM), or vehicle. Equal amounts of cellular protein (30 μg) were subjected to immunoblot analysis to detect phospho-IκBα, IκBα and β-actin. **f** Microglia were serum-starved for 30 min and then treated for 6 h with Plg (0.2 μM), Plg plus tPA (12 nM), LPS (0.1 μg/mL) or vehicle. RNA was isolated and RT-qPCR was performed to determine TNFα mRNA (mean ± SEM; *n* = 3; ***p < 0.001; one-way ANOVA with Tukey’s post hoc test. Statistical analysis is relative to the vehicle control). **g** Microglia were incubated for 1 h with Plg (0.2 μM), Plg plus tPA (12 nM), or LPS (0.1 μg/mL). Immunoblot analysis was performed to determine phospho-IκBα, total IκBα and β-actin. **h**–**j** N-astrocytes were serum-starved for 30 min and then treated with tPA alone (12 nM), Plg (0.2 μM) alone, or tPA plus Plg for 6 h. Expression of the mRNAs encoding TNFα, IL-1β, and CCL2 was determined (mean ± SEM; *n* = 3; ***p < 0.001; one-way ANOVA with Tukey’s post hoc test. Statistical analysis is relative to the vehicle control). **k** N-astrocytes were incubated for 1 h with tPA (12 nM), Plg (0.2 μM), or with tPA plus Plg. Immunoblot analysis was performed to detect phospho-IκBα, IκBα and β-actin. **l** N-astrocytes were treated with Plg (0.2 μM) for the indicated times. Immunoblot analysis was performed to detect phospho-IκBα, IκBα, and β-actin

N-astrocytes were treated with 12 nM tPA and 0.2 μM Plg for 6 h. Control cells were treated with LPS, which initiates cell-signaling and induces expression of pro-inflammatory cytokines in a TLR4-dependent manner [31], LTA, which activates TLR2 [32], or ODN 1826, which activates TLR9 [33]. RT-qPCR was performed to assess TNFα mRNA expression. GAPDH mRNA was determined as a normalizer. Figure 1a shows that Plg plus tPA robustly induced TNFα expression whereas the TLR agonists failed to have an effect. Similar results were obtained when we studied expression of CCL2 (Fig. 1b) and IL-1β (Fig. 1c). Because the role of GAPDH as a RT-qPCR normalizer for astrocytes has been called into question [34, 35], we repeated these studies using RPL13A and SDHA as normalizers. Highly similar results were obtained with both alternatives to GAPDH (see Additional file 1: Figure S1A–C). In control experiments, we studied mouse BMDMs. In these cells, Plg plus tPA induced expression of TNFα, as in N-astrocytes; however, so did all three TLR agonists, proving that the agonists were active (Fig. 1d).

The response of astrocytes to pro-inflammatory stimuli also was evaluated by examining IκBα phosphorylation, an index of NFκB activation [34]. N-astrocytes were treated with Plg plus tPA, LPS, LTA, or ODN 1826 for 1 h. In response to Plg plus tPA, IκBα was phosphorylated and the total abundance of IκBα was decreased (Fig. 1e), indicating NFκB activation. Responses were not observed with any of the TLR agonists. This result was confirmed by densitometry (see Additional file 1: Figure S1D).

### Plg activates N-astrocytes independently of exogenously added tPA

In BMDMs, induction of pro-inflammatory cytokine expression by Plg requires simultaneous addition of Plg activator and generation of catalytically active PM [21]. In experiments with cultured microglia, the same result was obtained. TNFα mRNA was robustly increased in cells treated with Plg plus tPA but not in cell treated with tPA or Plg alone (Fig. 1)f. The magnitude of the response of microglia to Plg plus tPA was similar to that observed with 0.1 μg/mL LPS, our positive control. IκBα was phosphorylated and decreased in total abundance in microglia treated with Plg and tPA but not in cells treated with tPA or Plg alone (Fig. 1g), again indicating that PM generation by exogenously added plasminogen activator is probably required to induce pro-inflammatory cytokine expression by these cells. The magnitude of the response to Plg plus tPA in microglia, as determined by densitometry analysis of the IκBα phosphorylation level and the decrease in total abundance of IκBα, was similar to that observed with LPS, as determined by densitometry (see Additional file 1: Figure S1E).

When N-astrocytes were treated with 200 nM Plg plus 12 nM tPA or with Plg in the absence of tPA for 6 h, TNFα mRNA expression was increased (Fig.1h). The magnitude of the effect was equivalent in the presence or absence of tPA. tPA alone failed to increase TNFα expression by N-astrocytes. Similar results were obtained when we studied expression of CCL2 (Fig. 1i) and IL-1ß (Fig. 1j). Again, expression of these pro-inflammatory cytokines was increased comparably by Plg plus tPA or by Plg alone. In the absence of Plg, tPA did not regulate expression of IL-1β or CCL2.

When N-astrocytes were treated with 0.2 μM Plg plus 12 nM tPA or with 0.2 μM Plg in the absence of tPA for 1 h, IκBα was phosphorylated and the total abundance of IκBα was decreased (Fig. 1k). tPA did not cause IκBα phosphorylation in the absence of Plg. This result was confirmed by densitometry (see Additional file 1: Figure S1F). Figure 1 l shows that in response to Plg, IκBα was phosphorylated gradually as a function of time, beginning at 45 min (see also Additional file 1: Figure S1G). This result is consistent with a model in which the Plg must be modified to initiate cell-signaling, for example by endogenously produced plasminogen activators.

### N-astrocytes activate Plg endogenously

**Fig. 2:**
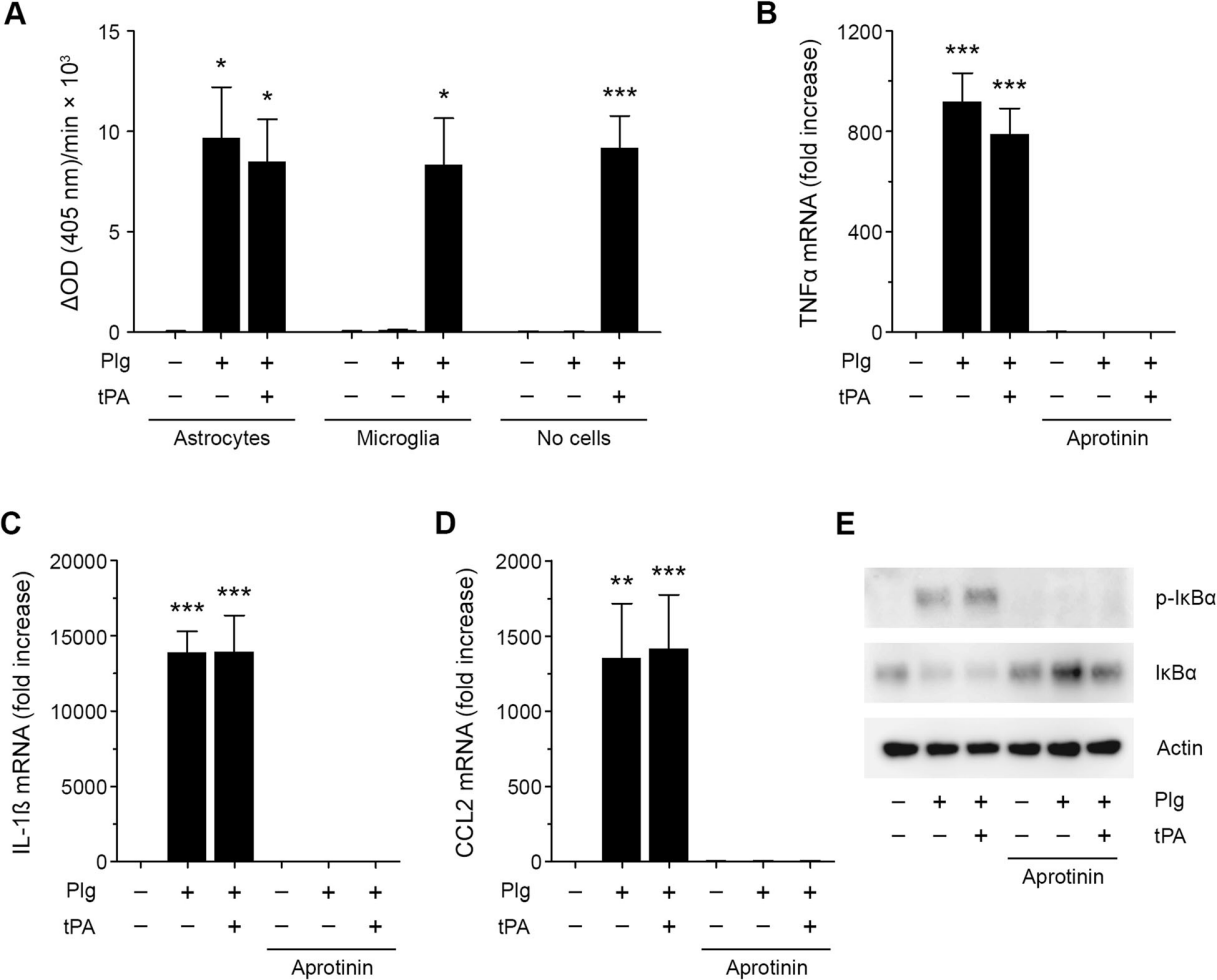
Plg activation is required for induction of cytokine expression by N-astrocytes. **a** Plg (0.2 μM), Plg plus tPA (12 nM) or vehicle was added to wells with N-astrocytes, microglia, or no cells for 6 h. Then, S-2251 (0.2 mM) was added. The velocity of S-2251 hydrolysis was determined ΔAbs 405 nm/min × 10^3^ (mean ± SEM, *n* = 3; **p < 0.01; *p < 0.1 one-way ANOVA with Tukey’s post hoc test. Statistical analysis is relative to the vehicle control). **b**–**d** N-astrocytes were treated for 6 h with Plg (0.2 μM), Plg plus tPA (12 nM) or vehicle, with and without Aprotinin (33 units/ml). RT-qPCR was performed to determine TNFα, IL-1β, and CCL2 mRNA (mean ± SEM; *n* = 3; ***p < 0.001, **p < 0.01; one-way ANOVA with Tukey’s post hoc test. Statistical analysis is relative to the vehicle control). **e** N-astrocytes were treated with Plg (0.2 μM), Plg plus tPA (12 nM), or vehicle in presence and absence of Aprotinin (33 units/ml) for 1 h. Immunoblot analysis was performed to detect phospho-IκBα, IκBα, and β-actin

To test whether N-astrocytes express endogenous plasminogen activators that allow Plg activation, we incubated 0.2 μM Plg with N-astrocytes, with microglia, and in wells without cells. In some wells, we added 12 nM tPA. After 6 h, PM was detected by measuring the rate of hydrolysis of S-2251. In N-astrocyte cultures, similar levels of PM were detected irrespective of whether tPA was added or not (Fig. 2a). By contrast, in wells with microglia, PM was detected only when tPA was added together with Plg. The same was true in wells without cells.

To confirm that Plg-induced cytokine expression in N-astrocytes requires PM generation, we treated N-astrocytes with Plg alone or Plg plus tPA for 6 h in the presence or absence of the plasmin active site inhibitor, aprotinin. Figures 2 b–d show that aprotinin blocked the effects of Plg and Plg plus tPA on expression of TNFα, IL-1β, and CCL2 by N-astrocytes. Aprotinin also blocked the ability of Plg and Plg plus tPA to induce IκBα phosphorylation in N-astrocytes (Fig. 2e, Additional file 1: Figure S1H).

### Plg receptors are necessary for induction of cytokine expression by Plg

**Fig. 3:**
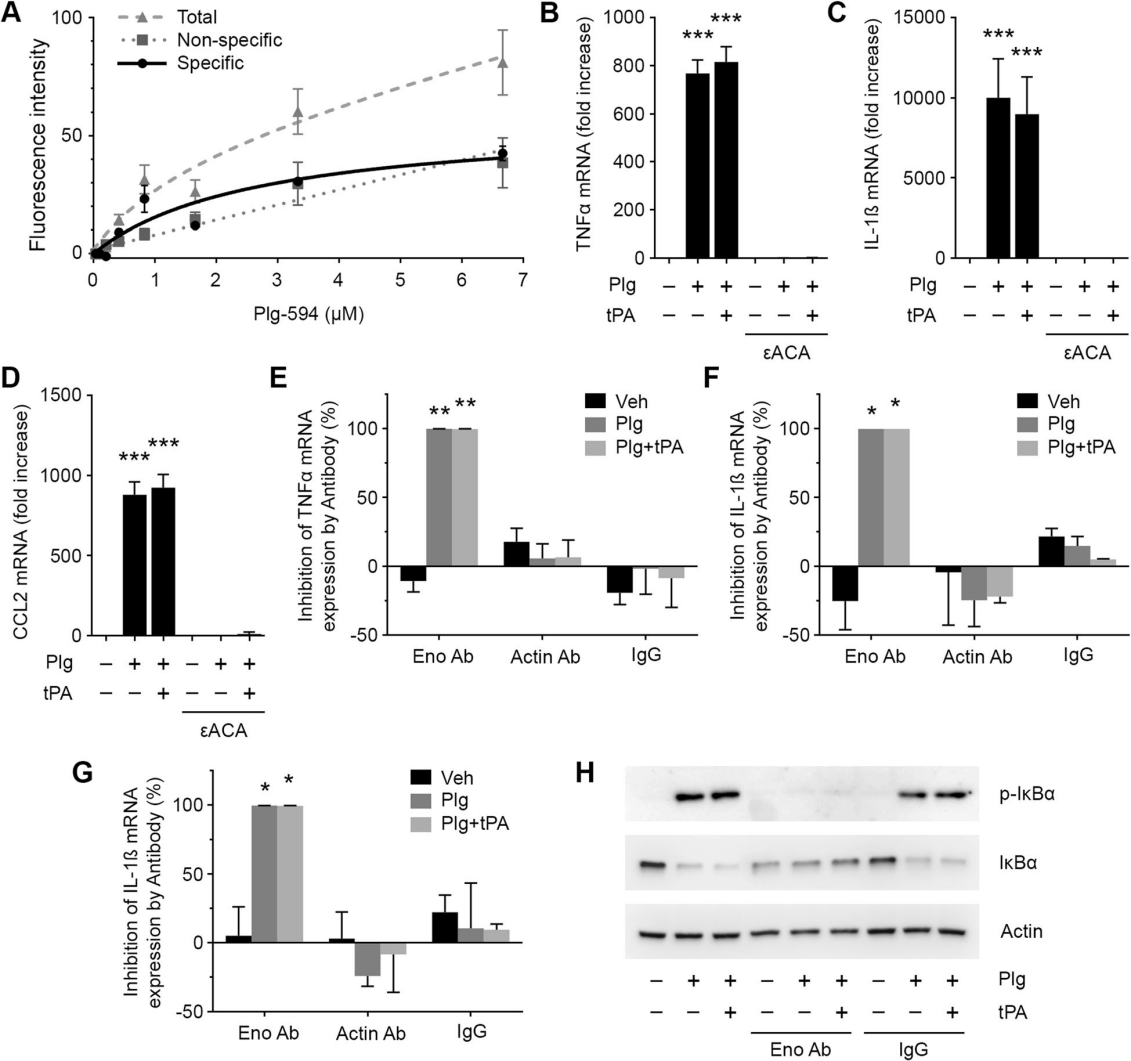
Plg receptors are required for induction of cytokine expression in N-astrocytes by Plg. **a** Specific binding of Plg-594 to N-astrocytes. The presented graph shows total binding, binding in the presence of εACA (non-specific), and specific binding, which is defined as binding that is displaced by εACA (mean of three separate binding studies). **b**–**d** N-astrocytes were treated with Plg (0.2 μM), Plg plus tPA (12 nM) or vehicle, in the presence or absence of 10 mM εACA for 6 h. RT-qPCR was performed to determine TNFα, IL-1β and CCL2 mRNA (mean ± SEM; *n* = 3; ***p < 0.001, **p < 0.01; one-way ANOVA with Tukey’s post hoc test. Statistical analysis is relative to the vehicle control). **e**–**g** N-astrocytes were treated with Plg (0.2 μM), Plg plus tPA (12 nM) or vehicle, in the presence of 10 μg/mL α-enolase-specific antibody, antibody that targets the Plg-binding site in actin (25 nM), or non-specific IgG for 6 h. RT-qPCR was performed to determine TNFα, IL-1β, and CCL2 mRNA. The ability of each antibody to inhibit cytokine expression in response to the eliciting agent (% inhibition) is shown (mean ± SEM; *n* = 4; **p < 0.01, *p < 0.05; two-way ANOVA with Tukey’s post hoc test). (**h**) N-astrocytes were treated with Plg (0.2 μM), Plg plus tPA (12 nM), or vehicle in presence and absence of α-enolase-specific antibody (10 μg/mL) for 1 h. Immunoblot analysis was performed to detect phospho-IκBα, IκBα, and β-actin

Plg receptors amplify the rate of Plg activation by tPA and uPA [14]. These receptors also may be involved in cell-signaling pathways activated by Plg [21, 35]. Because Plg receptor interactions are dependent on Plg lysine-binding sites, these interactions are competitively inhibited by εACA [30]. Figure 3a shows that N-astrocytes bound fluorescently labeled Plg (Plg-594) in a specific manner, when specific binding was defined by the fraction of bound Plg displaced by εACA. The K_D_ for specific binding of Plg-594 to N-astrocytes was 1.0 ± 0.3 μM, consistent with the known binding affinity of Plg to Plg receptors in other cell types [14]. When N-astrocytes were incubated with Plg alone or with Plg plus tPA, in the presence of εACA, expression of TNFα, IL-1β, and CCL2 were blocked (Fig. 3b–d). These results suggest that Plg receptors are required for Plg activation and for induction of pro-inflammatory cytokine expression by PM in N-astrocytes.

The molecular nature of Plg receptors varies from cell type to cell type. In many cell types, α-enolase, which is typically an intracytoplasmic protein, translocates to the cell surface and functions as a Plg receptor [36–38]. Like α-enolase, actin may translocate to the astrocyte cell-surface and has been reported to bind Plg and amplify Plg activation [39]. Figures 3 e–g show that, in N-astrocytes, α-enolase-specific antibody completely blocked expression of TNFα, IL-1β, and CCL2 in response to Plg or Plg plus tPA. Actin-specific antibody, by contrast, failed to inhibit cytokine expression. Non-specific IgG, at equivalent concentrations, also was without effect. α-enolase-specific antibody blocked the ability of Plg and Plg plus tPA to induce IκBα phosphorylation in N-astrocytes (Fig. 3h, Additional file 1: Figure S1I), supporting the results of our cytokine expression studies.

### PM induces inflammatory cytokine expression in N-astrocytes via a PAR-dependent pathway

**Fig. 4:**
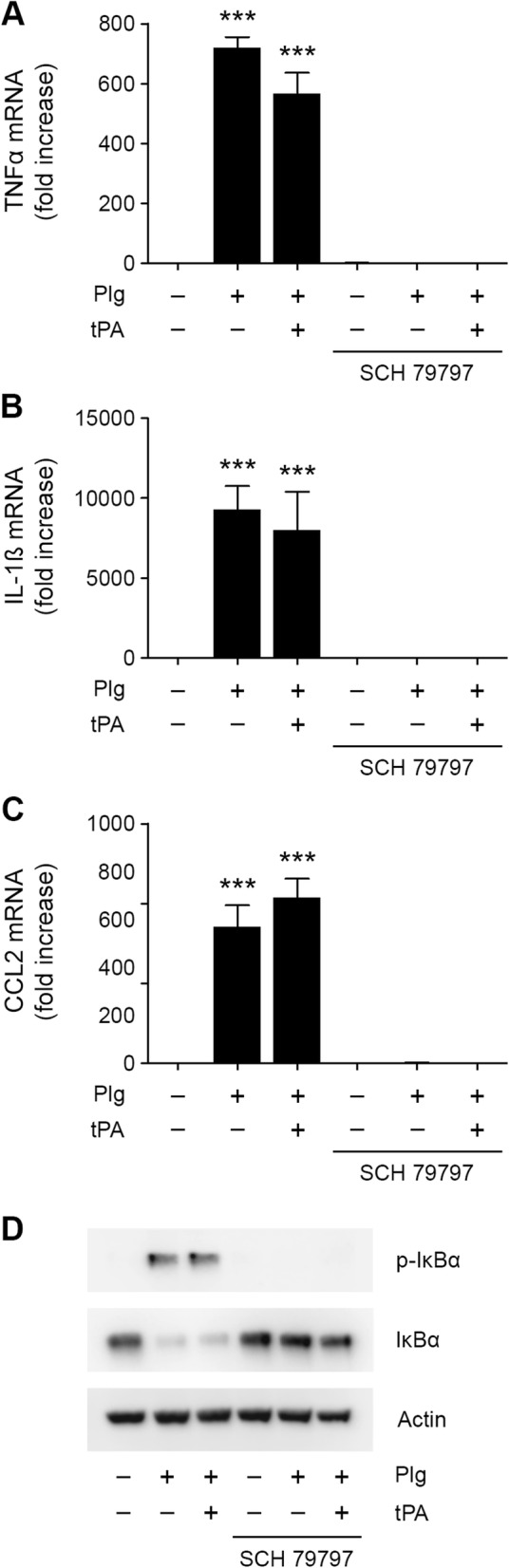
PAR-1 is essential in the pathway by which PM activates N-astrocytes. (**a**–**c**) N-astrocytes were treated with Plg (0.2 μM), Plg plus tPA (12 nM) or vehicle in presence and absence of SCH 79797 (2 μM) for 6 h. RT-qPCR was performed to determine expression of TNFα, IL-1β, and CCL2 (mean ± SEM; *n* = 3; ***p < 0.001; one-way ANOVA with Tukey’s post hoc test. Statistical analysis is relative to the vehicle control). **d** N-astrocytes were treated with Plg (0.2 μM), Plg plus tPA (12 nM), or with vehicle, in the presence and absence of SCH 79797 (2 μM) for 1 h. Immunoblot analysis was performed to detect phospho-IκBα, IκBα, and β-actin

PAR-1, a G-protein-coupled receptor that is activated by proteolytic cleavage, is strongly expressed in resting as well as reactive astrocytes and plays a key role in the astrocytic response to injury in vivo [40, 41]. To test whether PARs are involved in the pathway by which Plg induces cytokine expression in N-astrocytes, we treated these cells with the selective PAR-1 antagonist, SCH 79797. SCH 79797 completely blocked expression of TNFα, IL-1β, and CCL2 in response to Plg or Plg plus tPA (Fig. 4a–c). SCH 79797 also blocked IκBα phosphorylation when N-astrocytes were treated with Plg or Plg plus tPA for 1 h (Fig. 4d, Additional file 1: Figure S1J).

### Endogenously expressed uPA and uPAR are required for Plg-induced cytokine expression by N-astrocytes

**Fig. 5:**
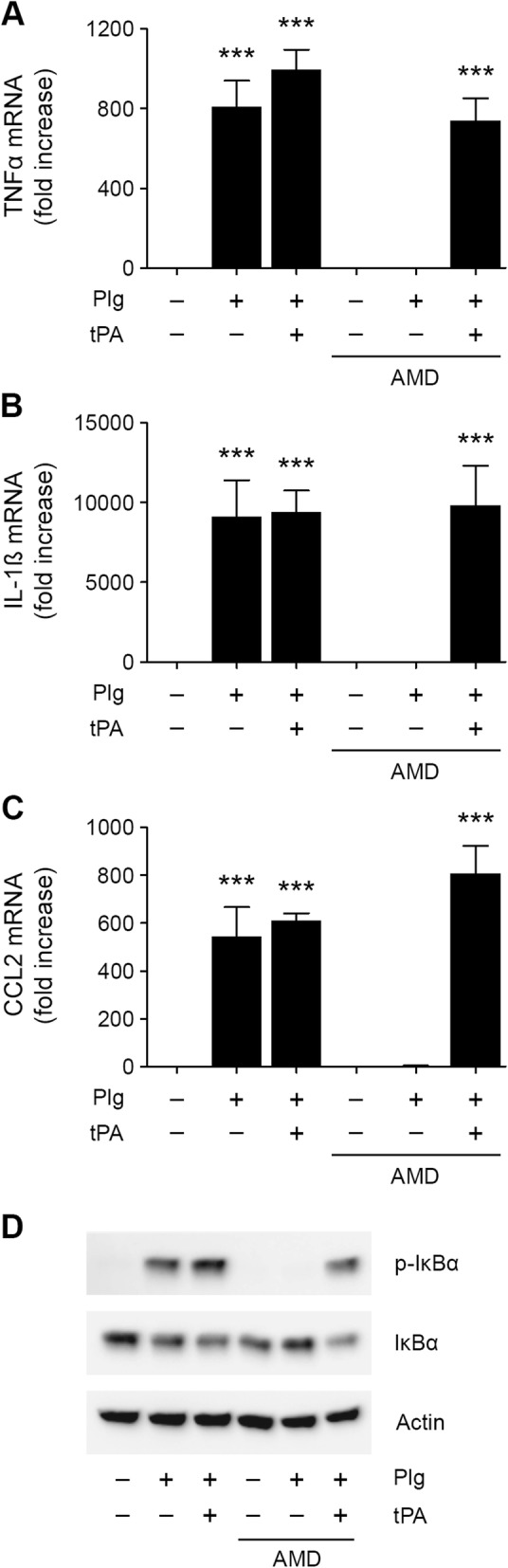
**a**–**d** N-astrocytes produce uPA to activated Plg endogenously. N-astrocytes were treated for 6 h with Plg (0.2 μM), Plg plus tPA (12 nM), or vehicle in the presence or absence of amiloride (AMD, 100 μM). RT-qPCR was performed to determine mRNA levels for TNFα, IL-1β, and CCL2 (mean ± SEM; *n* = 3; ***p < 0.001, **p < 0.01; one-way ANOVA with Tukey’s post hoc test). **d** N-astrocytes were treated with (0.2 μM), Plg plus tPA (12 nM), or vehicle in presence and absence of amiloride (100 μM) for 1 h. Immunoblot analysis was performed to detect phospho-IκBα, IκBα, and β-actin

AMD is a specific inhibitor of uPA enzymatic activity that does not affect the activity of tPA [42]. To identify the plasminogen activator expressed by N-astrocytes and responsible for converting Plg into an astrocyte-activating agent, cells were incubated with Plg, Plg plus tPA, or vehicle in presence or absence of AMD. AMD blocked expression of TNFα, IL-1β, and CCL2 in N-astrocytes treated with Plg alone, but not in cells treated with Plg plus tPA (Fig. 5a–c). These results suggest that the endogenous Plg activator in N-astrocytes is uPA. In IκBα phosphorylation studies, AMD had the same effect, inhibiting the response to Plg alone but not to Plg plus tPA (Fig. 5d, Additional file 1: Figure S1K).

**Fig. 6:**
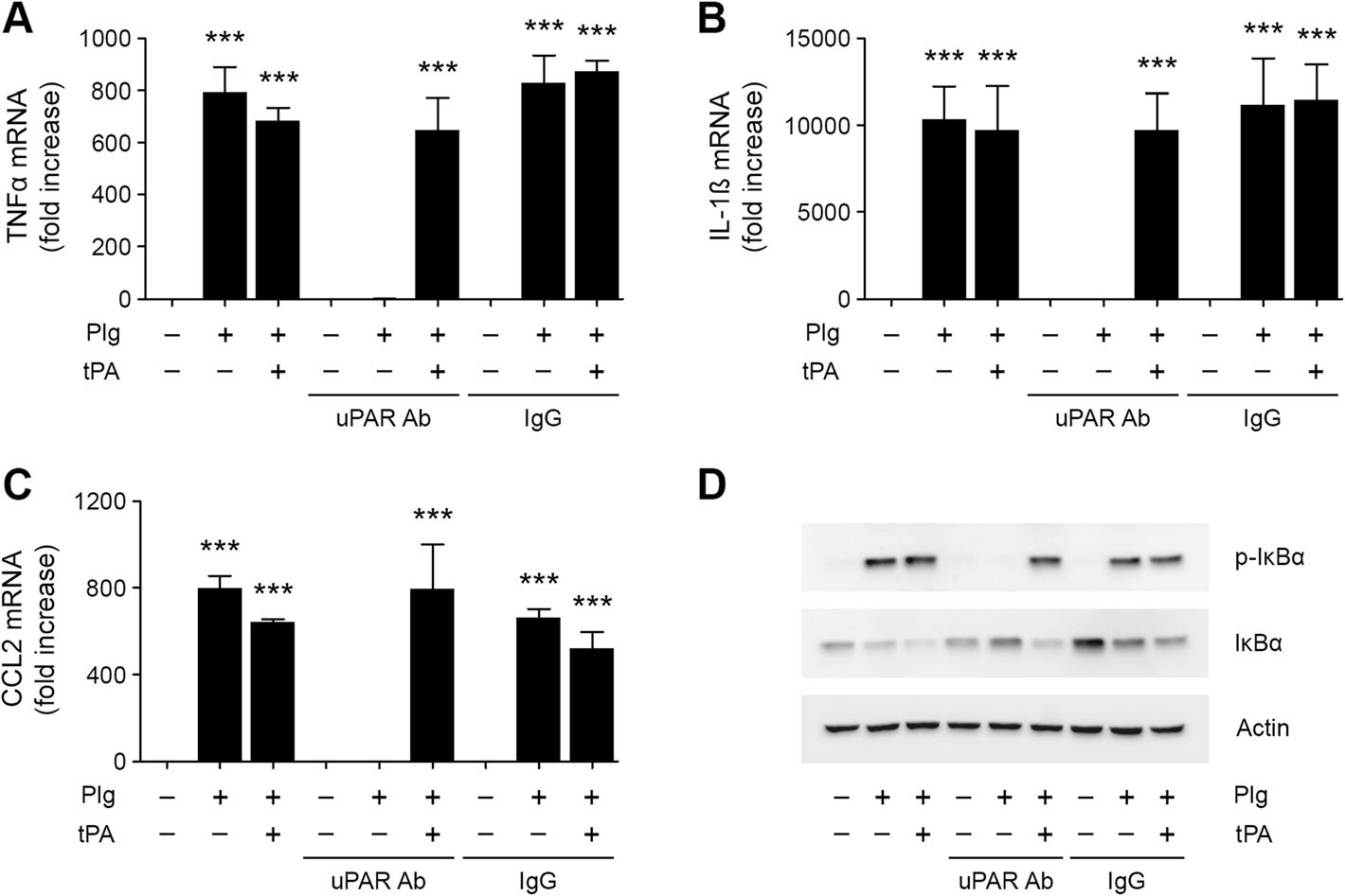
uPAR-specific antibody neutralizes the effects of Plg on cytokine expression by N-astrocytes. (**a**–**c**) N-astrocytes were treated with Plg (0.2 μM), Plg plus tPA (12 nM), or vehicle in presence and absence of uPAR-specific antibody (1 μg/mL) or an equivalent concentration of non-specific IgG for 6 h. Expression of TNFα, IL-1β, and CCL2 was determined by RT-qPCR (mean ± SEM; *n* = 3; ***p < 0.001; one-way ANOVA with Tukey’s post hoc test. Statistical analysis is relative to the vehicle control). **d** N-astrocytes were treated with Plg (0.2 μM), Plg plus tPA (12 nM) or vehicle in presence and absence of uPAR-specific antibody (1 μg/mL) or an equivalent concentration of non-specific IgG. Immunoblot analysis was performed to detect phospho-IκBα, IκBα and β-actin

uPAR promotes Plg activation by uPA and also directly triggers cell-signaling in response to uPA [15]. To test whether uPAR is necessary in the pathway by which Plg induces pro-inflammatory cytokine expression by N-astrocytes, we treated N-astrocytes with Plg alone, Plg plus tPA, or vehicle in the presence of uPAR-specific antibody or non-specific IgG. uPAR-specific antibody neutralized the effects of Plg on expression of TNF-α, IL-1β, and CCL2 but did not inhibit cytokine expression in response to Plg plus tPA (Fig. 6a–c). Non-specific IgG was without effect. uPAR-specific antibody also inhibited IKBα phosphorylation selectively in response to Plg alone but not Plg plus tPA (Fig. 6d, Additional file 1: Figure S1L). Thus, uPAR plays an essential role in the pathway by which Plg stimulates pro-inflammatory cytokine expression in N-astrocytes in the absence of exogenously added Plg activator.

### The uPA ATF inhibits Plg-induced N-astrocyte activation

**Fig. 7:**
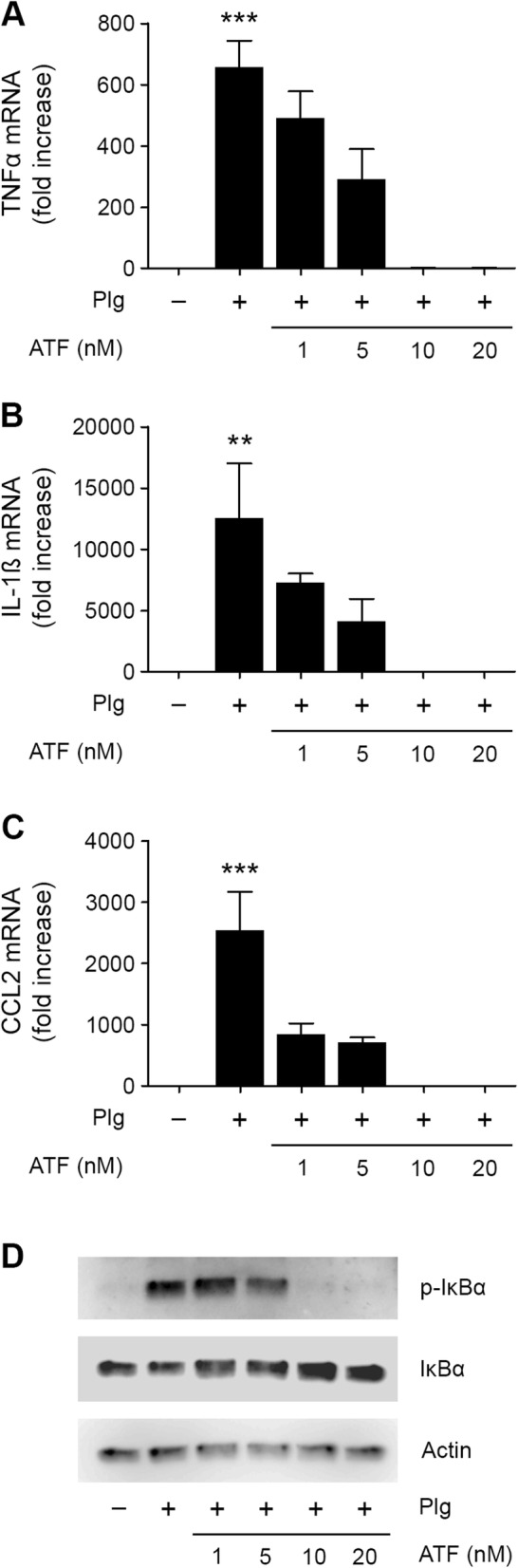
The uPA ATF blocks cytokine expression by N-astrocytes in response to Plg. **a**–**c** N-astrocytes were treated for 6 h with Plg (0.2 μM) and the indicated concentrations of uPA ATF. Expression of TNFα, IL-1β, and CCL2 was determined by RT-qPCR (mean ± SEM; *n* = 3; ***p < 0.001; one-way ANOVA with Tukey’s post hoc test. Statistical analysis is relative to the vehicle control). **d** N-astrocytes were treated for 1 h with Plg (0.2 μM) and the indicated concentrations of uPA ATF. Immunoblot analysis was performed to detect phospho-IκBα, IκBα, and β-actin

The uPA ATF binds to uPAR, similarly to full-length uPA, and reproduces the signaling responses elicited when uPA binds to uPAR [24–26]. To determine whether uPAR functions in promoting Plg-induced cytokine expression by N-astrocytes by facilitating Plg activation or through uPAR-initiated cell-signaling, we treated N-astrocytes with Plg in the presence of increasing concentrations of uPA ATF. Figures 7 a–c show that the uPA ATF inhibited the effects of Plg on expression of TNFα, IL-1β, and CCL2; the activity of the uPA ATF was ATF-concentration-dependent. The uPA ATF also inhibited IκBα phosphorylation in N-astrocytes in response to Plg (Fig. 7d, Additional file 1: Figure S1M). These results suggest that the principal activity of uPAR is to facilitate Plg activation by endogenously produced uPA and not to trigger cell-signaling. uPA ATF probably functions by competitively displacing uPA from uPAR, which disrupts the Plg activation receptor complex.

## Discussion

Neuro-inflammation is increasingly recognized as an important regulator of diverse neurological diseases, including multiple sclerosis, Alzheimer’s disease, other forms of neurodegeneration, traumatic brain injury, ischemic stroke, and central sensitization in pain processing [1, 2, 43–46]. While the role of microglia in neuro-inflammation is well recognized, astrocytes also are major contributors, especially in chronic CNS inflammatory states, despite their more limited repertoire of TLRs and other PRRs [1, 2, 5, 6].

There is considerable evidence that activation of fibrinolysis may contribute to neuro-inflammation. When LPS is injected into the hippocampus of Plg gene knock-out mice, the inflammatory response within the brain is attenuated [47]. In experimental autoimmune encephalomyelitis (EAE), an accepted model of multiple sclerosis, Plg gene knock-out mice develop delayed disease of lesser severity [48]. Furthermore, depleting Plg outside the CNS, using antisense oligonucleotide (ASO) technology, decreases the severity of pathology in a mouse model of Alzheimer’s Disease [49]. In this latter study, complex mechanisms may be operational because the ASO approach does not appear to affect Plg levels within the CNS. Although Plg is expressed mainly by the liver, other cells and tissues, including CNS cells, express Plg [50].

The role of uPA and uPAR in neuro-inflammation appears more context-specific. Expression of uPA in the CNS is increased in neuro-inflammation [51]. Furthermore, in ischemic brain injury, binding of uPA expressed by neurons to astrocytic uPAR results in uPAR-initiated cell-signaling and astrocytic activation [23]. However, uPA and uPAR appear to be protective in EAE [52], as opposed the exacerbating role that would be predicted if the major role of uPA and uPAR is Plg activation [48].

**Fig. 8:**
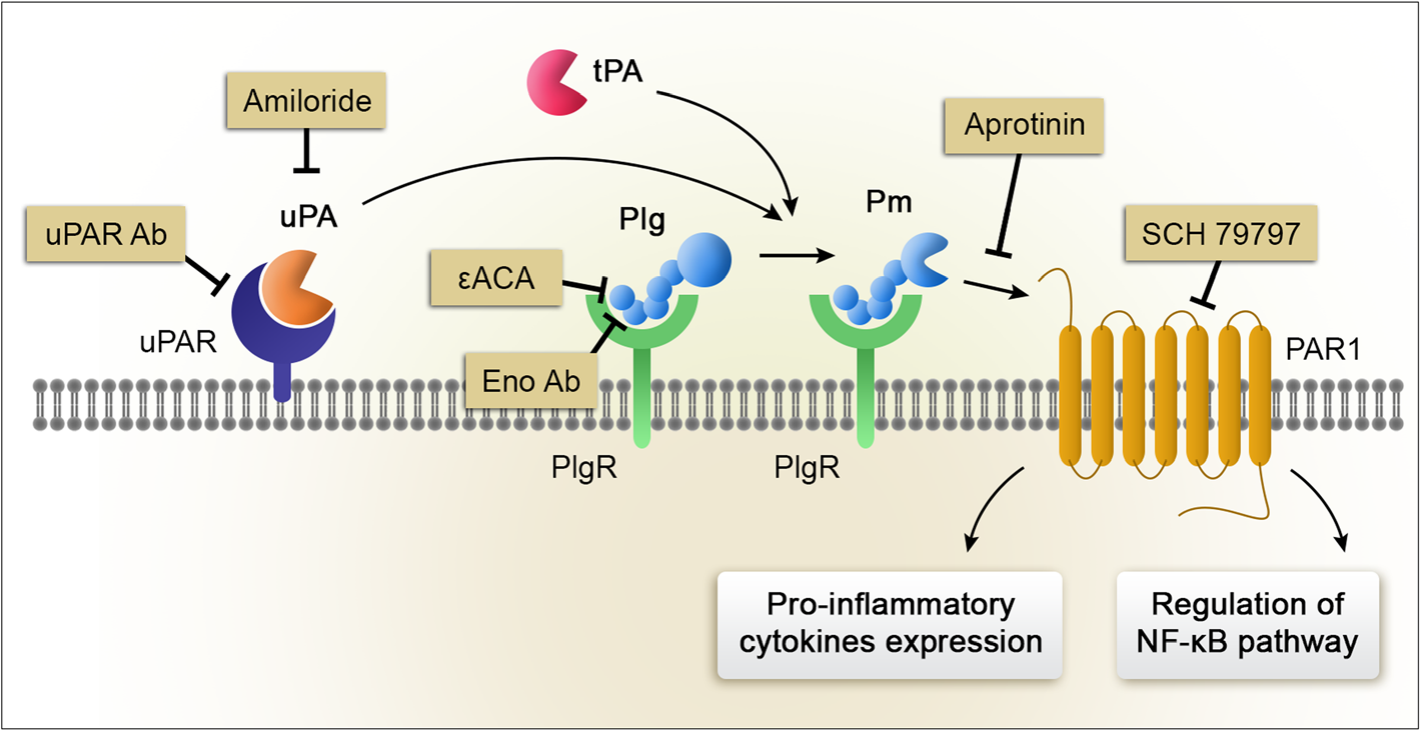
Model showing the orchestrated action of fibrinolysis receptors in the induction of cytokine expression by Plg in N-astrocytes. Multiple targets for antagonizing this pathway are identified

The results presented herein demonstrate that PM generation induces expression of the potently pro-inflammatory cytokines, TNFα, IL-1β, and CCL2, in microglia and astrocytes. In N-astrocytes, the pathway by which Plg increases cytokine expression is tightly controlled by a series of fibrinolysis receptors, including the Plg receptor, α-enolase, uPAR, and PAR-1. α-enolase and uPAR appear to function mainly to amplify Plg activation, implicating PAR-1 as the major signal transduction initiator. The ability of the ATF to block cytokine expression by Plg in N-astrocytes, suggests that uPAR-initiated cell-signaling is not sufficient in and of itself to activate these astrocytes. Our results with the uPA ATF do not rule out the possibility that when N-astrocytes produce uPA endogenously, uPAR-associated uPA has the dual role of promoting Plg activation, which is essential for cytokine induction, and triggering uPAR signaling, which may regulate the degree of astrocytic activation, as previously observed [23]. Figure 8 presents a model showing how three distinct fibrinolysis receptors work in concert to promote astrocytic activation in response to Plg. Multiple targets for antagonizing this pathway are identified.

N-astrocytes differed from microglia in culture and from cultured BMDMs, studied previously [21], in that Plg-induced cytokine expression independently of exogenously added Plg activator. This result reflects the ability of N-astrocytes in culture to express uPA and activate Plg in the absence of added tPA. In intact brain, astrocytic expression of uPA may be highest during development and then decrease [53]. However, in a tissue, as opposed to cell culture, astrocytic uPAR has the capacity to ligate uPA derived from any cell type in the cellular microenvironment. Thus, in various forms of pathology, the pathway described here may become operational due to astrocytic loading of uPAR with uPA produced by other cell types such as uPA [23].

N-astrocytes also differed from microglia and cultured BMDMs in that these cells demonstrated limited responsiveness to TLR agonists. In our studies, N-astrocytes failed to express increased levels of pro-inflammatory cytokines in response to agonists for TLR2, TLR4, and TLR9. Farina et al. showed that human astrocytes express TLR3 [7]. These same investigators were unable to demonstrate astrocytic expression of TLR2, TLR4, or TLR9. However, others have shown that expression of some TLRs may be increased when astrocytes are activated [54, 55]. Understanding how the activity of well described pattern recognition receptors and fibrinolysis receptors are integrated in the function of astrocytes in innate immunity is an important topic for future work.

LDL Receptor-related Protein-1 (LRP1) is a tPA receptor not studied here; however, its activity in promoting tPA endocytosis by astrocytes is previously reported [56]. Based on its ability to clear tPA from the astrocytic cell-surface, LRP1 has the capacity to regulate the amount of tPA available for Plg activation. LRP1 also may regulate the abundance of cell-surface uPAR via endocytosis [57]. Either of these mechanisms may regulate astrocytic activation and expression of pro-inflammatory cytokines by astrocytes in vivo.

Our results identifying α-enolase and not actin as a cell-surface Plg receptor in astrocytes contrast with previous results supporting a role for actin [39]. An important difference in the two studies; however, may be the major plasminogen activator considered. In the previous study, actin was identified as essential in promoting Plg activation by tPA. In the pathway by which Plg leads to cytokine expression, studied here, uPA appeared to be the essential Plg activator. Although actin-specific antibody did not inhibit cytokine expression in our experiments in which cells were treated with Plg plus tPA, it is still possible that distinct Plg receptors function in astrocytes to promote Plg activation by tPA or uPA. PAR-1 is best known for its function as a thrombin receptor, which informs cells that coagulation has been activated; however, PAR-1 is proteolytically activated by other proteases as well, including plasmin [58, 59]. In monocytes and macrophages, PM-mediated PAR cleavage is pro-inflammatory [18–21]. We concluded that a PAR and probably PAR-1 is the ultimate signal transducer responsible for the response to PM in N-astrocytes based on studies with the PAR-1-specific inhibitor, SCH 79797. This reagent completely blocked expression of TNFα, IL-1β, and CCL2 in response to Plg. PAR-1 may be a target for inhibiting neuro-inflammation. Because the pathway by which Plg causes pro-inflammatory cytokine expression by N-astrocytes is linear, in addition to PAR-1, the other two required fibrinolysis receptors in this pathway, α-enolase and uPAR, also may be targets for inhibiting the activity of PM. The effectiveness of targeting uPAR was demonstrated here in studies with uPAR-specific antibody and the uPA ATF. Hypothetically, opposing astrocytic activation may attenuate the crosstalk with microglia and other CNS cells, which adds to the chronicity of inflammation.

## Conclusions

In the CNS, activation of astrocytes leads to crosstalk with cells such as microglia and contributes to chronic neuro-inflammation. Compared with microglia, astrocytes express a less robust continuum of Pattern Recognition Receptors. We describe a pathway by which the zymogen form of the fibrinolysis protease, plasminogen, causes astrocytic activation and expression of pro-inflammatory cytokines, including TNF-α, IL-1β, and CCL2. The pathway requires a series of receptors for fibrinolysis proteins, including α-enolase, uPAR, and PAR-1. Targeting any of these receptors on astrocytes blocked the pro-inflammatory activity of plasminogen. Fibrinolysis protease receptors may be targets for inhibiting neuro-inflammation.

## Supplementary information

**Additional file 1: Figure S1.** (A-C) N-astrocytes were serum-starved for 30 min and then treated for 6 h with Plg alone or with Plg (0.2 μM) plus tPA (12 nM), or with vehicle. Expression of the mRNAs encoding TNFα, IL-1β and CCL2 was determined using RPL13A or SDHA as qPCR normalizers. (D-M) Densitometric analysis of the immunoblots presented in the paper.

## Data Availability

Not applicable

## Abbreviations

AMD: Amiloride
ATF: Amino terminal fragment of uPA
BMDMs: Bone Marrow-derived macrophages
CCL2: Chemokine (C-C motif) ligand 2
εACA: ε-Aminocaproic acid
GAPDH: Glyceraldehyde 3-phosphate dehydrogenase
GPI: Glycosylphosphatidylinositol
IL-1β: Interleukin-1β
LPS: Lipopolysaccharide
LTA: Lipoteichoic acid
NFκB: Nuclear Factor kappa-light-chain-enhancer of activated B cells
NMDA-R: N-methyl-D-aspartate Receptor
ODN: Oligodeoxynucleotide
PAR: Protease-activated Receptor
PL: Plasmin
Plg: Plasminogen
TLRs: Toll-Like receptors
TNFα: Tumor necrosis factor-α
tPA: Tissue-type plasminogen activator
uPA: Urokinase-type plasminogen activator
