# Supplementary figures and images for: Fibrinolysis protease receptors promote activation of astrocytes to express pro-inflammatory cytokines
Source: J Neuroinflammation. 2019 Dec 6;16:257. doi: 10.1186/s12974-019-1657-3 (PMC6896679; doi:10.1186/s12974-019-1657-3)

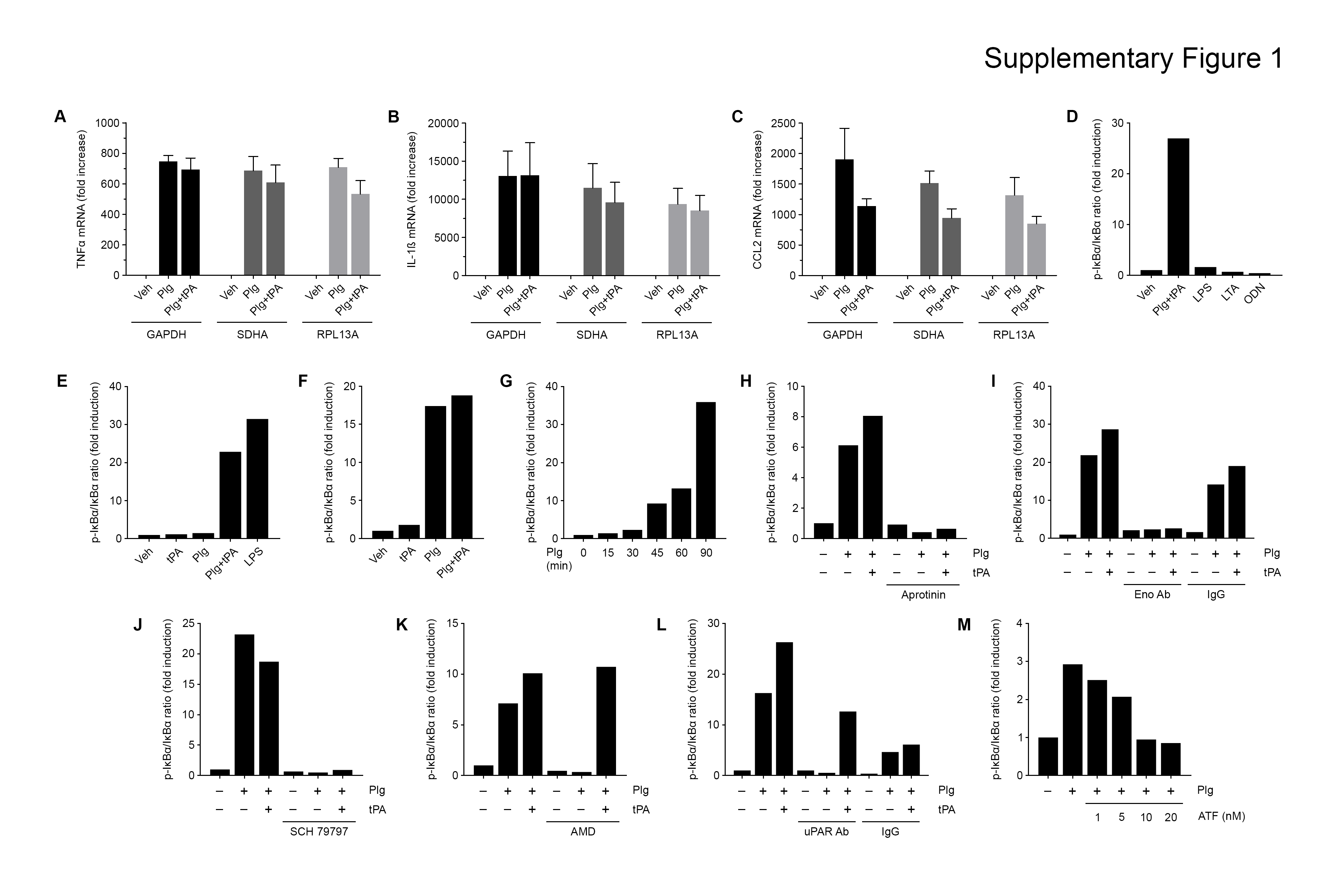

Supplement: Supplementary file 1 — Additional file 1: Figure S1. (A-C) N-astrocytes were serum-starved for 30 min and then treated for 6 h with Plg alone or with Plg (0.2 μM) plus tPA (12 nM), or with vehicle. Expression of the mRNAs encoding TNFα, IL-1β and CCL2 was determined using RPL13A or SDHA as qPCR normalizers. (D-M) Densitometric analysis of the immunoblots presented in the paper. [file 12974_2019_1657_MOESM1_ESM.tif]
